# Prevalence and Influencing Factors of Anxiety and Depression Symptoms in the First-Line Medical Staff Fighting Against COVID-19 in Gansu

**DOI:** 10.3389/fpsyt.2020.00386

**Published:** 2020-04-29

**Authors:** Juhong Zhu, Lin Sun, Lan Zhang, Huan Wang, Ajiao Fan, Bin Yang, Wei Li, Shifu Xiao

**Affiliations:** ^1^Department of Psychiatry, Lanzhou University Second Hospital, Lanzhou, China; ^2^Department of Geriatric Psychiatry, Shanghai Mental Health Center, Shanghai Jiao Tong University School of Medicine, Shanghai, China; ^3^Alzheimer's Disease and Related Disorders Center, Shanghai Jiao Tong University, Shanghai, China

**Keywords:** COVID-19, anxiety, depression, medical staff, Chinese

## Abstract

**Background:**

The outbreak of novel coronavirus pneumonia (COVID-19) has brought enormous physical and psychological pressure on Chinese medical staff. It is extremely important to understand the prevalence and influencing factors of anxiety and depression symptoms in first-line anti-epidemic medical staff and their coping styles for these negative emotions.

**Methods:**

A cross-sectional survey was conducted in Gansu (China), with a questionnaire packet which consisted of the self-rating anxiety scale (SAS), self-rating depression scale (SDS), and the simplified coping style questionnaire (SCSQ). A total of 79 doctors and 86 nurses participated in the survey. Correlation analysis was performed to explore the relationship between SAS, SDS, and SCSQ score. A linear regression model was used to determine the influencing factors for anxiety or depression symptoms.

**Results:**

The prevalence rates of anxiety and depression symptoms among doctors was 11.4% and 45.6%, respectively. History of depression or anxiety (T=-2.644, p= 0.010, 95%CI: -10.514~-1.481) was shown to be a risk factor for anxiety symptoms in doctors, while being male (T=2.970, p=0.004, 95%CI: 2.667~13.521) was a protective factor for depression. The prevalence rate of anxiety and depression symptoms among nurses was 27.9% and 43.0%, respectively. History of depression or anxiety was a common risk factor for anxiety symptoms (T=-3.635, p=0.000, 95%CI: -16.360~-4.789) and depression symptoms (T=-2.835, p=0.005, 95%CI:-18.238~-3.254) in nurses. The results of partial correlation analysis (controlled for gender and history of depression or anxiety) indicated that the total score of positive coping was negatively correlated with the total score of anxiety (r=-0.182, p=0.002) and depression (r=-0.253, p=0.001).

**Conclusions:**

The first-line anti-epidemic medical staff have high anxiety and depression symptoms and adopting positive coping styles will help to improve their negative emotions.

## Introduction

Since mid-December of 2019, coronavirus disease 2019 (COVID-19) has been identified in many countries (1). As of February 24^th^, 41600 medical staff in China have been fighting in the front line of the anti-epidemic campaign. Of these medical staff, 3387 of them were infected with the novel coronavirus pneumonia, accounting for 4% of all confirmed cases, while 22 died and accounted for 0.8% of the deaths. The front-line medical staff will not only bear the work pressure of overload, but also face the huge risk of infection (2). Stress represents the main environmental risk factor for psychiatric illnesses, and in a long-term stress state, people can be more prone to depression or other mental disease (3), which will also increase the risk of infection (4–6). Therefore, it is necessary to investigate the psychological state of the first-line anti-epidemic medical staff and give them necessary psychological interventions if they have anxiety or depression.

## Materials and Methods

### Participants

This cross-sectional study was conducted between February 1, 2020 and February 29, 2020. The research objects were the first line medical staff in the designated hospitals and fever clinics of novel coronavirus pneumonia in Gansu Province. The inclusion criteria were as follows: 1) 18 years or older; 2) doctor or nurse; 3) first-line to COVID-19; 4) without serious mental illness, such as schizophrenia or an intellectual disability; 5) without physical disease affecting anxiety or depression, such as hypothyroidism or coronary heart disease; and 6) willing to be investigated. Exclusion criteria were as follows: 1) less than 18 years old, 2) non-frontline medical or administrative staff, 3) not in Gansu, 4) serious mental illness or a combination of disorders that may affect anxiety or depression, and 5) refused to be investigated. Finally, 165 front-line medical staff working in Gansu were enrolled in the study.

Ethical approval was issued by the Ethics Committee of the Second Clinical Medical College of Lanzhou University, and all the participants had signed an informed consent before the study was initiated.

### Investigation Tools

By using the self-designed questionnaire, we have obtained the general demographic information of the respondents, including gender, age, marriage, occupation, education level, specialty, level of worry, and level of expectation.

### Neuropsychological Tests

The self-rating anxiety scale (SAS) (7) and self-rating depression scale (SDS) (8) were used to assess anxiety and depression symptoms of medical staff respectively. Both SAS and SDS are 20-item Likert and norm-referenced scales, in which items tap physiological and psychological symptoms and are rated by participants according to how each applied to them within the past week, using a 4-point scale ranging from 1 (none, or a little of the time) to 4 (most, or all of the time) (9). The choice of SAS items is based on diagnostic criteria listed in the major American psychiatry literature, whereas the SDS taps depressive symptoms based on factor analytic studies of depression symptoms (9). The Zung Self-Rating Anxiety/Depression Scale (SAS/SDS) has been widely applied in clinical institutions and scientific research, showing convincing results and a remarkable degree of consensus among clinicians (10). According to the conclusion of other studies (11), we also utilized the standard score of 50 as the critical value to divide depression or anxiety in the present study. The Simplified Coping Style Questionnaire (SCSQ) (12) is a 20-item self-report scale that includes two dimensions. The first entry consists of 1–12 items and reflects the traits of passive coping and active coping (8-item). The second entry consists of 13–20 questions, reflecting the traits of passive coping. This instrument has been commonly used in China and has proven to be highly reliable and valid (13).

### Investigation Way

In the current study, we used the Electronic “Questionnaire Star” Questionnaire as the survey tool, and information was collected through friend circle forwarding and WeChat group promotion. “Questionnaire Star” is a professional online survey platform, which can be used for questionnaire surveys, evaluation, voting, and other purposes. Compared with traditional survey methods, “Questionnaire Star” has the obvious advantages of being fast, low cost, easy to learn, and easy to use (14).

### Statistical Analysis

The categorical variables were expressed as the frequency (%), while the continuous variables were presented as mean± SD. A single sample Kolmogorov-Smirnov test was used to test whether the data conform to normal distribution. Chi square test was used to compare categorical variables, while independent sample t-test and Mann-whitney U test were respectively used to compare the continuous variables with and without normal distribution. The prevalence of anxiety and depression symptoms was calculated by splitting the total number of cases diagnosed by the total number of participants. The influence factors of anxiety or depression were analyzed by linear regression analysis. Partial correlation analysis was used to analyze the correlation between SCSQ and SDS/SAS. The statistical analysis was performed by using SPSS version 22.0 and a p-value < 0.05 was regarded as significant.

## Results

### General Demographic Data of Frontline Medical Staff

Through the online survey from Questionnaire Stars, we finally received 165 qualified questionnaires, and the recovery rate of the questionnaire was 100%. The average age of the respondents was 34.16 ± 8.06 (years), the average length of employment was 11.35 ± 8.60 (years), the average working day on the fever clinic or isolation ward was 15.65 ± 46.63 (days), the average anxiety score was 42.79 ± 8.50, and the average depression score was 46.94 ± 11.60. **Tables 1** and **2** show the results.

**Table 1:** General demographic information of medical staff.

Variables	N=165
Age, y	34.16 ± 8.06
Length of employment, y	11.35 ± 8.60
Working days in epidemic area, d	15.65 ± 46.63
Total anxiety score	42.79 ± 8.50
Total depression score	46.94 ± 11.60
Occupation	
Doctor,n(%)	79(47.9)
Nurse,n(%)	86(52.1)
Gender	
Male,n(%)	28(17)
Female,n(%)	137(83)
Marriage	
Married,n(%)	39(23.6)
Unmarried,n(%)	119(72.1)
Divorce or other,n(%)	7(4.2)
Got kids	
Yes,n(%)	112(67.9)
No,n(%)	53(32.1)
Only child or not	
Yes,n(%)	32(19.4)
No,n(%)	133(80.6)
Education	
Secondary specialized school,n(%)	10(6.1)
Junior College,n(%)	43(26.1)
Undergraduate,n(%)	100(60.6)
Master,n(%)	11(6.7)
Doctor and above,n(%)	1(0.6)
History of anxiety or depression	
Yes,n(%)	23(13.9)
No,n(%)	142(86.1)
Family history of anxiety or depression	
Yes,n(%)	11(6.7)
No,n(%)	154(93.3)
Specialty	
Department of respiration,n(%)	12(7.3)
Department of Infectious disease,n(%)	29(17.6)
Intensive Care Unit,n(%)	2(1.2)
Others,n(%)	122(73.9)

**Table 2:** The medical staff's most troublesome concerns and prevalent expectations.

Variables	N=165
Objects of concern	
Heavy workload, n(%)	12(7.3)
Lack of professional knowledge, n(%)	65(39.4)
Can't go home for a long time, n(%)	23(13.9)
Inconvenient operation in protective clothing, n(%)	10(6.1)
Be infected, n(%)	55(33.3)
Expected content	
Get together with family as soon as possible, n(%)	14(8.5)
Rotation of medical staff, n(%)	8(4.8)
Psychosocial support, n(%)	4(2.4)
Leadership care, n(%)	7(4.2)
An early end to the epidemic, n(%)	132(80.0)
Total score of active response	33.53 ± 6.74
Total score of negative response	17.45 ± 4.28

### Prevalence and Influencing Factors of Anxiety and Depression Symptoms (Doctors)

With 50 points as the critical value (both for SAS and SDS), nine people were considered to have anxiety symptoms, with a prevalence of 11.4% (9/79), and 36 people were considered to have depression symptoms, with a prevalence of 45.6% (36/79). There were statistical differences (p < 0.05) in the total score of negative responses and a history of anxiety or depression between the anxiety group and non -anxiety group, while there were statistical differences (p < 0.05) in gender between the depression group and non –depression group. **Table 3** presents the results.

**Table 3:** Possible influencing factor related to anxiety or depression in doctors.

Variables	Anxiety (n=9)	Non-anxiety (n=70)	X^2^ or t	p
Total score of negative response	20.56 ± 4.85	17.30 ± 4.54	2.010	0.048*
History of anxiety or depression				
Yes,n(%)	4(44.4)	10(14.3)	4.975	0.047*
No,n(%)	5(55.6)	60(85.7)		
Variables	Depression (n=36)	Non-depression (n=43)	X^2^ or t	P
Gender				
Male,n(%)	7(19.4)	21(48.8)	7.399	0.009*
Female,n(%)	29(80.6)	22(51.2)		

*means p < 0.05.

Then, by using linear regression analysis, we found that history of depression or anxiety (T=-2.644, p= 0.010, 95%CI: -10.514~-1.481) was a risk factor for anxiety symptoms in doctors, while being male (T=2.970, p=0.004, 95%CI: 2.667~13.521) was a protective factor for depression. **Table 5** lists the results.

### Prevalence and Influencing Factors of Anxiety and Depression Symptoms (Nurses)

With 50 points as the critical value (both for SAS and SDS), 24 people were considered to have anxiety symptoms, with a prevalence of 27.9% (24/86), and 37 people were considered to have depression symptoms, with a prevalence of 43.0% (37/86). There were statistical differences (p < 0.05) in the history of anxiety or depression between the anxiety group and non -anxiety group, while there were statistical differences (p < 0.05) in the total scores of active response, history of anxiety or depression, and specialty between the depression group and non–depression group. **Table 4** lists the results.

**Table 4:** Possible influencing factor related to anxiety or depression in nurses.

Variables	Anxiety (n=24)	Non-anxiety (n=62)	X^2^ or t	p
History of anxiety or depression				
Yes,n(%)	6(25.0)	3(4.8)	7.506	0.013*
No,n(%)	18(75.0)	59(95.2)		
Variables	Depression (n=37)	Non-depression (n=49)	X^2^ or t	P
Total score of active response	32.65 ± 6.107	35.22 ± 5.843	-1.985	0.050*
History of anxiety or depression				
Yes,n(%)	7(18.9)	2(4.1)	4.953	0.035*
No,n(%)	30(78.1)	47(95.9)		
Specialty				
Department of respiration, n(%)	7(18.9)	1(2.0)	12.240	0.007*
Department of Infectious disease,n(%)	5(13.5)	20(40.8)		
Intensive Care Unit,n(%)	1(2.7)	1(2.0)		
Others,n(%)	24(64.9)	27(55.1)		

*means p < 0.05.

**Table 5:** Linear regression analysis for anxiety and depression in doctors.

Variables	B	S.E	beta	T	p	95%confidence interval	
						lower limit	Upper limit
Anxiety							
History of anxiety or depression	-5.998	2.268	-0.289	-2.644	0.010*	-10.514	-1.481
Depression							
Male	8.094	2.725	0.321	2.970	0.004*	2.667	13.521

*means p < 0.05; The total score of anxiety was taken as the dependent variable and the variable with differences in table 3 as the independent variable.

Then, by using linear regression analysis, we found that a history of depression or anxiety was a common risk factor for anxiety symptoms (T=-3.635, p=0.000, 95%CI: -16.360~-4.789) and depression symptoms (T=-2.835, p=0.005, 95%CI:-18.238~-3.254) in nurses. **Table 6** lists the results.

**Table 6:** Linear regression analysis for anxiety and depression in nurses.

Variables	B	S.E	beta	T	p	95%confidence interval	
						lower limit	Upper limit
Anxiety							
History of anxiety or depression	-10.574	2.909	-0.369	-3.635	<0.001*	-16.360	-4.789
Depression							
History of anxiety or depression	-10.746	3.767	-0.297	-2.853	0.005*	-18.238	-3.254

*means p < 0.05; The total score of anxiety was taken as the dependent variable and the variable with differences in table 5 as the independent variable.

### The Relationship Between Coping Styles and Anxiety and Depression

The results of partial correlation analysis (controlled for gender and history of depression or anxiety) indicated that the total score of positive coping was negatively correlated with the total score of anxiety (r=-0.182, p=0.002) and depression (r=-0.253, p=0.001)

## Discussion

In the current study, we investigated the prevalence and influencing factors of anxiety and depression symptoms in the first-line medical staff fighting against novel coronavirus pneumonia in Gansu, and came to some interesting conclusions: 1) the prevalence rate of anxiety and depression symptoms among doctors was 11.4% and 45.6%, respectively; 2) the prevalence rate of anxiety and depression symptoms among nurses was 27.9% and 43.0%, respectively; 3) a history of depression or anxiety (T=-2.644, p= 0.010, 95%CI: -10.514~-1.481) was a risk factor for anxiety symptoms in doctors, while being male (T=2.970, p=0.004, 95%CI: 2.667~13.521) was a protective factor for depression; 4) a history of depression or anxiety was a common risk factor for anxiety symptoms (T=-3.635, p=0.000, 95%CI: -16.360~-4.789) and depression symptoms (T=-2.835, p=0.005, 95%CI:-18.238~-3.254) in nurses; 5) the results of partial correlation (controlled for gender and history of depression or anxiety) indicated that the total score of positive coping was negatively correlated with the total score of anxiety (r=-0.182, p=0.002) and depression (r=-0.253, p=0.001).

Previous studies had suggested that the mental health status of medical staff was worse than in the general population (15, 16). For example, Kerrien M et al. (17) found that 27% of junior doctors were suffering from depression, while 28.7% were suffering from anxiety. Paiva CE et al. (18) found that 12.3%of doctors had depression (HADS-D ≥ 11), and 19.4% had anxiety (HADS-A ≥ 11). A systematic review of 29 studies showed that the prevalence of anxiety and depression among students in medical schools in Europe, the UK, and elsewhere in the English-speaking world outside of North America was 7.7-65.5% and 6.0-66.5%, respectively (19). Similarly, a cross-sectional survey showed that the prevalence of anxiety symptoms in nurses was 43.4% (20). Another cross-sectional survey indicated that the prevalence rate of anxiety among female nurses was 41.1% (21). Maharaj S et al. (22) found that the prevalence rates of depression and anxiety among Australian nurses were 32.4% and 41.2%, respectively. So, our conclusions were not consistent, and this difference might come from the use of different investigation tools. Interestingly, we found that the anxiety level (X^2^ = 7.019. p=0.011) of nurses was significantly higher than that of doctors, but there was no difference in depression (X^2^ = 0.108. p=0.756) between the two groups. So we need to pay more attention to nurses in order to relieve their anxiety.

In the present study, we found that being male (T=2.970, p=0.004, 95%CI: 2.667~13.521) was a protective factor for depression in doctors. There is considerable discourse surrounding the disproportionate diagnosis of men with depression as compared to women, often times cited at a rate around 1:2 (23). However, the view that depression rates are universally higher in women is challenged, as biological determinants, sex role changes, and social factors might also contribute to this difference (24). What's more, meta-analyses of diagnoses and symptoms show that the gender difference peaks in adolescence but then will decline and remain stable in adulthood, and cross-national analyses also indicate that larger gender differences are found in nations with greater gender equity for major depression, but not depression symptoms (25). But beyond that, a systematic review of nineteen studies indicates that the male gender is significantly associated with suicide in individuals with depression (26). So, the relationship between gender and depressive symptoms needs to be verified by a large-scale longitudinal study.

We also found that a history of anxiety or depression was a risk factor for anxiety symptoms in doctors, and a common risk factor for anxiety and depression symptoms in nurses. Osasona SO et al. (27) found that previous mental illness was significantly associated with anxiety, depression, or general psychiatric morbidity in a sample of inmates in a Nigerian prison. Ahmed A et al. (28) found that a history of depression and stress was associated with depressive and anxiety symptoms in a sample of 615 women in Saskatchewan from pregnancy to 5 years postpartum. Stafford L et al. (29) found that women with a psychiatric history and high neuroticism are at the greatest risk for future morbidity after adjusting for confounders, such as age, education, and living alone. Therefore, our conclusions are consistent.

By using partial correlation analysis (controlled for gender and history of depression or anxiety), we finally found that a positive coping style was a protective factor for anxiety(r=-0.182, p=0.002) and depression (r=-0.253, p=0.001), suggesting that a positive coping style was helpful in resisting negative emotions. Mahmoud JS et al. (30) found that reducing maladaptive coping behaviors might have the most positive impact on reducing anxiety, depression, and stress in young adult college students. Wang Y et al. (31) found that perceived stress had some positive effects on psychological distress, and coping style might be a mediator in this relationship among Chinese physicians. In addition, Holz NE et al. (32) pointed out that stress exposure would increase rates of depression and anxiety in adults, particularly in females, and had been associated with maladaptive changes in the anterior cingulate cortex (ACC), while positive coping styles could help to increase the ACC volume. So our conclusions were consistent.

We have to admit that our research has certain limitations. First, it was just a cross-sectional study that could not establish a causal link. Second, the sample size used was relatively small and therefore reduced the reliability of the study. Third, we used the self-assessment scale to evaluate the depression and anxiety symptoms of the medical staff, which might have a certain result deviation.

## Conclusions

The first-line anti-epidemic medical staff have high anxiety and depression symptoms, and adopting a positive coping style will help to improve their negative emotions.

## Data Availability Statement

The datasets analyzed in this article are not publicly available because the datasets are part of an unpublished database and the database is still being used for other manuscripts in preparation. Requests to access the datasets should be directed to 156892477@qq.com.

## Ethics Statement

The studies involving human participants were reviewed and approved by the Ethics Committee of the Second Clinical Medical College of Lanzhou University. The patients/participants provided their written informed consent to participate in this study. Written informed consent was obtained from the individual(s) for the publication of any potentially identifiable images or data included in this article.

## Author Contributions

WL and LS contributed to the study concept and design. JZ acquired the data. LZ, HW, and AF collected the data. SX and BY analyzed the data and drafted the manuscript. All authors read and approved the final manuscript.

## Funding

This work was supported by grants from the China Ministry of Science and Technology (2009BAI77B03), National Natural Science Foundation of China (number 81671402), Clinical research center project of Shanghai Mental Health Center (CRC2017ZD02), the National Key R&D program of China (2017YFC1310501500), the Cultivation of Multidisciplinary interdisciplinary Project in Shanghai Jiaotong University (YG2019QNA10), and curriculum reform of Medical College of Shanghai Jiaotong University.

## Conflict of Interest

The authors declare that the research was conducted in the absence of any commercial or financial relationships that could be construed as a potential conflict of interest.
