# Corrigendum: Prevalence and Influencing Factors of Anxiety and Depression Symptoms in the First-Line Medical Staff Fighting Against COVID-19 in Gansu

**DOI:** 10.3389/fpsyt.2021.653709

**Published:** 2021-03-24

**Authors:** Juhong Zhu, Lin Sun, Lan Zhang, Huan Wang, Ajiao Fan, Bin Yang, Wei Li, Shifu Xiao

**Affiliations:** ^1^Department of Psychiatry, Lanzhou University Second Hospital, Lanzhou, China; ^2^Department of Geriatric Psychiatry, Shanghai Mental Health Center, Shanghai Jiao Tong University School of Medicine, Shanghai, China; ^3^Alzheimer's Disease and Related Disorders Center, Shanghai Jiao Tong University, Shanghai, China

**Keywords:** COVID-19, anxiety, depression, medical staff, Chinese

In the original article, there were several errors. In the **Introduction** section, the sentences “Since mid-December of 2019, coronavirus disease 2019 (COVID-19) has been spreading from China (Wuhan) to 26 countries worldwide (1). As of February 24th, 41600 medical staff across the country have been fighting in the front line of the anti-epidemic campaign.” was inaccurate. In the **Materials and Methods** section, in the last sentence of the first paragraph of the Participants subsection, “165 front-line medical staff working in the Gansu” were incorrectly written as medical staff working in the Wuhan isolation ward. In the **Results** section, table numbers in the second paragraph of the Prevalence and Influencing Factors of Anxiety and Depression Symptoms (Doctors) subsection and the first paragraph of the Prevalence and Influencing Factors of Anxiety and Depression Symptoms (Nurses) were cited incorrectly.

The fully corrected paragraphs are as below:

The **Introduction** section:

“Since mid-December of 2019, coronavirus disease 2019 (COVID-19) has been identified in many countries (1). As of February 24th, 41600 medical staff in China have been fighting in the front line of the anti-epidemic campaign. Of these medical staff, 3387 of them were infected with the novel coronavirus pneumonia, accounting for 4% of all confirmed cases, while 22 died and accounted for 0.8% of the deaths. The front-line medical staff will not only bear the work pressure of overload, but also face the huge risk of infection (2). Stress represents the main environmental risk factor for psychiatric illnesses, and in a long-term stress state, people can be more prone to depression or other mental disease (3), which will also increase the risk of infection (4–6). Therefore, it is necessary to investigate the psychological state of the first-line anti-epidemic medical staff and give them necessary psychological interventions if they have anxiety or depression.”

The **Materials and Methods** section, the **Participants** subsection, paragraph 1:

“This cross-sectional study was conducted between February 1, 2020 and February 29, 2020. The research objects were the first line medical staff in the designated hospitals and fever clinics of novel coronavirus pneumonia in Gansu Province. The inclusion criteria were as follows: (1) 18 years or older; (2) doctor or nurse; (3) first-line to COVID-19; (4) without serious mental illness, such as schizophrenia or an intellectual disability; (5) without physical disease affecting anxiety or depression, such as hypothyroidism or coronary heart disease; and (6) willing to be investigated. Exclusion criteria were as follows: (1) less than 18 years old, (2) non-frontline medical or administrative staff, (3) not in Gansu, (4) serious mental illness or a combination of disorders that may affect anxiety or depression, and (5) refused to be investigated. Finally, 165 front-line medical staff working in Gansu were enrolled in the study.”

The **Results** section, the **Prevalence and Influencing Factors of Anxiety and Depression Symptoms (Doctors)** subsection, paragraph 2:

“Then, by using linear regression analysis, we found that history of depression or anxiety (T = −2.644, *p* = 0.010, 95% CI: −10.514~−1.481) was a risk factor for anxiety symptoms in doctors, while being male (T = 2.970, *p* = 0.004, 95% CI: 2.667~13.521) was a protective factor for depression. **Table 5** lists the results.

The **Results** section, the **Prevalence and Influencing Factors of Anxiety and Depression Symptoms (Nurses)** subsection, paragraph 1:

With 50 points as the critical value (both for SAS and SDS), 24 people were considered to have anxiety symptoms, with a prevalence of 27.9% (24/86), and 37 people were considered to have depression symptoms, with a prevalence of 43.0% (37/86). There were statistical differences (*p* < 0.05) in the history of anxiety or depression between the anxiety group and non-anxiety group, while there were statistical differences (*p* < 0.05) in the total scores of active response, history of anxiety or depression, and specialty between the depression group and non–depression group. **Table 4** lists the results.”

In the original article, the first reference was incorrectly written as “1. Lim J, Jeon S. Case of the Index Patient Who Caused Tertiary Transmission of COVID-19 Infection in Korea: the Application of Lopinavir/Ritonavir for the Treatment of COVID-19 Infected Pneumonia Monitored by Quantitative RT-PCR. *J Korean Med Sci* (2020) 35(6):e79. doi: 10.3346/jkms.2020.35.e88.” It should be “1. World Health Organization. *WHO Director-General's Remarks at the Media Briefing on COVID-19 Outbreak on 18 February 2020* (2020). Available online at: https://www.who.int/director-general/speeches/detail/who-director-general-s-remarks-at-the-media-briefing-on-covid-19-outbreak-on-18-february-2020 (access February 20, 2020).”

The authors apologize for this error and state that this does not change the scientific conclusions of the article in any way. The original article has been updated.

